# Use of oral corticosteroids in patients with asthma: how far is clinical practice from the guidelines? Results from surveys of patients and doctors

**DOI:** 10.3389/falgy.2025.1638304

**Published:** 2025-10-13

**Authors:** Luisa Brussino, Gianna Camiciottoli, Annalisa Stassaldi, Diego Bagnasco, Simona Barbaglia, Maria Beatrice Bilò, Cristiano Caruso, Filippo Cipriani, Renato Cutrera, Giuliana Nicolosi, Jasmine Nour, Giulia Scioscia, Giorgio Walter Canonica

**Affiliations:** 1 Dipartimento di Scienze Mediche, Università Degli Studi di Torino—SCDU Immunologia e Allergologia, AO Ordine Mauriziano di Torino, Turin, Italy; 2 Department of Clinical and Biomedical Sciences “Mario Serio”, University of Florence—Severe Asthma Unit, Careggi University Hospital, Florence, Italy; 3 Sanofi, Milan, Italy; 4 Respiratory and Allergy Clinic, IRCCS Ospedale Policlinico San Martino, Genoa, Italy; 5 Department of Internal Medicine (DIMI), University of Genoa, Genoa, Italy; 6 Presidente Associazione Nazionale Pazienti RESPIRIAMO INSIEME-APS, Padova, Italy; 7 Department of Clinical and Molecular Science (DISCLIMO), Università Politecnica Delle Marche—Allergy Unit, Azienda Ospedaliero-Universitaria delle Marche, Ancona, Italy; 8 UOSD Allergy and Clinical Immunology, Fondazione Policlinico A. Gemelli IRCCS, Rome, Italy; 9 Pneumology and Cystic Fibrosis Unit, ‘Bambino Gesù’ Children’s Hospital, IRCCS, Rome, Italy; 10 Department of Medical and Surgical Sciences, University of Foggia, Foggia, Italy; 11 Personalized Medicine, Asthma and Allergy, Humanitas Clinical and Research Center, IRCCS, Rozzano, Italy; 12 Department of Biomedical Sciences, Humanitas University, Pieve Emanuele, Milan, Italy

**Keywords:** adherence to therapy, asthma management, multidisciplinary approach, oral corticosteroids (OCS), OCS dependency, patient-centered care, severe asthma, treatment guidelines

## Abstract

**Introduction:**

Asthma is often treated with oral corticosteroids (OCS), despite their association with significant adverse effects. While guidelines recommend minimizing OCS use through alternative therapies and patient-centered approaches, discrepancies between recommendations and real-world practices persist. This study evaluates OCS usage patterns and barriers to adherence to asthma treatment guidelines in Italy, using surveys conducted with healthcare professionals (HCPs) and patients.

**Methods:**

Two cross-sectional surveys were administered between January and March 2024 to HCPs and asthma patients. The surveys assessed OCS prescription practices, treatment adherence, patient involvement, adverse event management, and perceptions of OCS use. Descriptive analysis was performed to identify patterns and highlight gaps in current practices.

**Results:**

The surveys revealed considerable variability in OCS prescribing practices, treatment duration and daily dosages. Over 80% of patients reported using OCS and 18% of HCPs believed that the maximum daily doses of OCS are higher than the guideline-recommended doses. Patients did not feel fully involved in treatment decisions, with over 40% of patients reporting unsatisfactory communication about treatment alternatives or adverse effects. Barriers to optimal care included inadequate access to specialists, inconsistent monitoring protocols, and a lack of multidisciplinary approaches. Both HCPs and patients highlighted the need for clearer definitions of OCS dependency and enhanced tools for tracking treatment adherence.

**Discussion:**

The findings underscore the urgent need for systemic reforms to align clinical practice with guidelines. These include establishing pragmatic definitions for OCS dependency, promoting multidisciplinary care, and leveraging technology for monitoring. Addressing psychosocial factors and empowering patients through education and shared decision-making are also critical.

## 1 Introduction

Asthma affects approximately 262 million people worldwide, with severe asthma (SA) representing a significant burden despite affecting only a small percentage of patients (1). Asthma incidence peaks in children under 9 years old (2). According to estimates from the Global Asthma Network, asthma affected 9.1% of children, 11.0% of adolescents and 6.6% of adults worldwide in 2022 (3). Asthma prevalence has risen by 15% globally between 1990 and 2019, with new cases on the rise in Western Sub-Saharan Africa and other regions (2, 4). In Italy, among the estimated 4 million asthma patients, about 200,000 have SA and account for a disproportionate share of healthcare resources and costs (5, 6).

The management of SA often requires the use of inhaled corticosteroids (ICS), long-acting β2-agonists (LABAs), and long-acting muscarinic antagonists (LAMAs). While oral corticosteroids (OCS) are used either as short-term courses for severe exacerbations or as long-term therapy for uncontrolled SA, guidelines recommend their use only as a last resort due to severe adverse effects (7, 8). These effects include iatrogenic adrenal insufficiency, growth impairment in children, and increased mortality risk compared to no use or periodic use (9–13).

Despite the availability of targeted biologic drugs for SA, and clear guidelines limiting OCS use, OCS medications remain widely prescribed in asthma treatment (14–16). Patient perception of OCS is often negative, with studies showing that 44% of adult asthma patients have concerns about OCS use, leading some to reduce or discontinue treatment without medical supervision by healthcare professionals (HCPs) (17).

To address the gap between the guidelines and clinical practice, and rectify the lack of published information on discrepancies between real-life treatment and guideline recommendations in the Italian context, the Respiriamo Insieme Association (a non-profit organization for respiratory diseases) in collaboration with Sanofi conducted two surveys—one among HCPs and another among patients in Italy. This article presents the results of these surveys, aiming to highlight the discrepancies and suggest strategies to reduce OCS dependence while improving quality of life for individuals living with SA.

## 2 Materials and methods

### 2.1 Design

Two cross-sectional surveys were undertaken in Italy targeting (1) specialist HCPs (hereafter referred to as Survey 1), and (2) asthma patients (Survey 2). Both questionnaires were developed by a multidisciplinary team; the Sanofi medical department developed Survey 1, and members of the scientific committee of Respiriamo Insieme developed Survey 2. Participation was anonymous and voluntary; consent was considered to be implicit upon agreement to participate. There were no associated costs or incentives offered to participants. Both surveys, conducted between January and March 2024, were completed online and took approximately 5–10 min each. Data analysis was primarily descriptive, reporting proportions.

#### 2.1.1 Survey 1—healthcare professionals

Survey 1 was distributed via clickable links to pulmonologists and allergists/immunologists included in a proprietary database maintained by Sanofi Italia. The survey aimed to gather insights into the prescribing practices of these specialists in relation to OCS use in patients with SA, exploring potential overuse patterns, barriers to proper use, and alignment with current guidelines. The questionnaire consisted of seven closed-ended questions (six multiple-choice questions with single selection and one rating scale). Initial invitations were sent via email, with a follow-up email sent 2 weeks later to initial non-respondents.

#### 2.1.2 Survey 2—patients

The second survey aimed to gather insights into OCS use from the patient perspective. This survey was accessible online to all asthma patients residing in Italy and receiving care through the Italian National Health Service. The questionnaire consisted of 13 closed-ended questions and was disseminated through Respiriamo Insieme's database, as well as through its social media channels. The survey aimed to understand patients' perspectives on OCS use, including: (1) usage patterns and prescription adherence; (2) patient awareness and involvement in treatment decisions; (3) medical guidance and education provided by HCPs; (4) treatment adjustment and monitoring; (5) multidisciplinary care and management of adverse events (AEs); and (6) self-medication and safety practices related to OCS use.

### 2.2 Ethics and privacy

Participation in the survey was voluntary and consent was implicit. As this study investigated patient and physician opinions, ethical approval was not required. All participant data were anonymized for analysis and processed in compliance with European Regulation EU 2016/679.

## 3 Results

### 3.1 Survey 1—healthcare professionals

**Figure 1:**
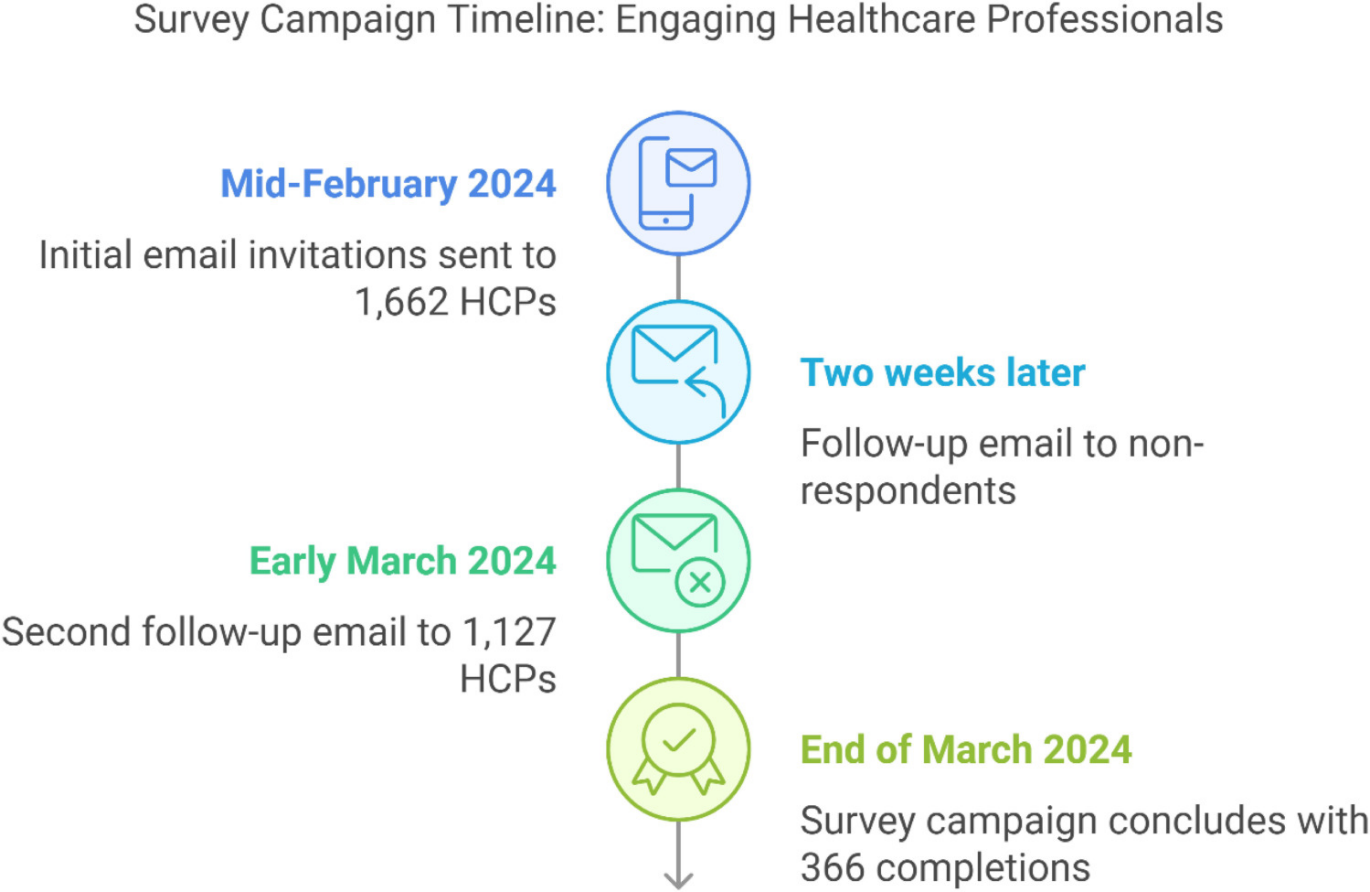
HCP survey (Survey 1) flow and participation rates. HCP, healthcare professional.

Of the 1662 HCPs who were sent the email invitation, 698 HCPs (42%) opened the survey email, 283 (17%) clicked the survey link and 197 completed the survey (12% completion rate). A second follow-up email was sent in early March to 1,127 HCPs (Figure 1); 508 HCPs (45%) opened the follow-up email, 189 (17%) clicked the survey link and 169 HCPs completed the survey (14% completion rate). In total, 366 surveys were completed.

#### 3.1.1 S1.Q1: optimal duration of oral corticosteroid treatment

**Figure 2:**
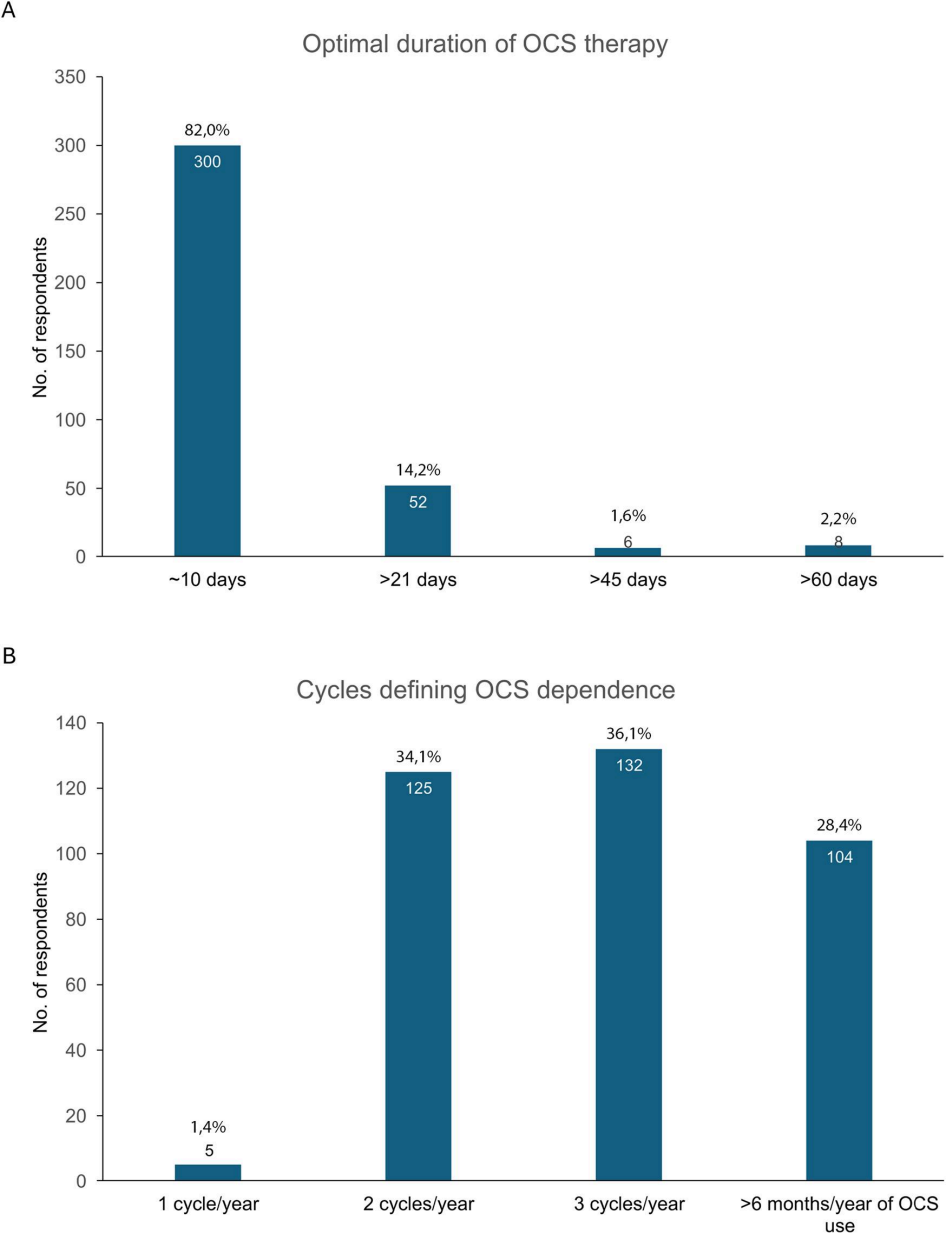
**(A)** Preferred duration of OCS treatment and **(B)** criteria for OCS dependency in Survey 1. OCS, oral corticosteroids.

The preferred duration of OCS treatment for most of the HCPs (82%) was 10 days, while 14% preferred a duration of more than 21 days (Figure 2A). The remaining HCPs opted for longer treatment durations.

#### 3.1.2 S1.Q2: criteria for defining oral corticosteroid dependency

When asked to determine the number of OCS cycles required to classify a patient as OCS-dependent, 99% of HCPs answered either 2 or 3 cycles per year or more than 6 months of OCS use per year. Interestingly, 1% of the respondents considered that a single cycle was enough to consider the patient as OCS dependent (Figure 2B).

#### 3.1.3 S1.Q3: calculating annual cumulative oral corticosteroid dose

**Figure 3:**
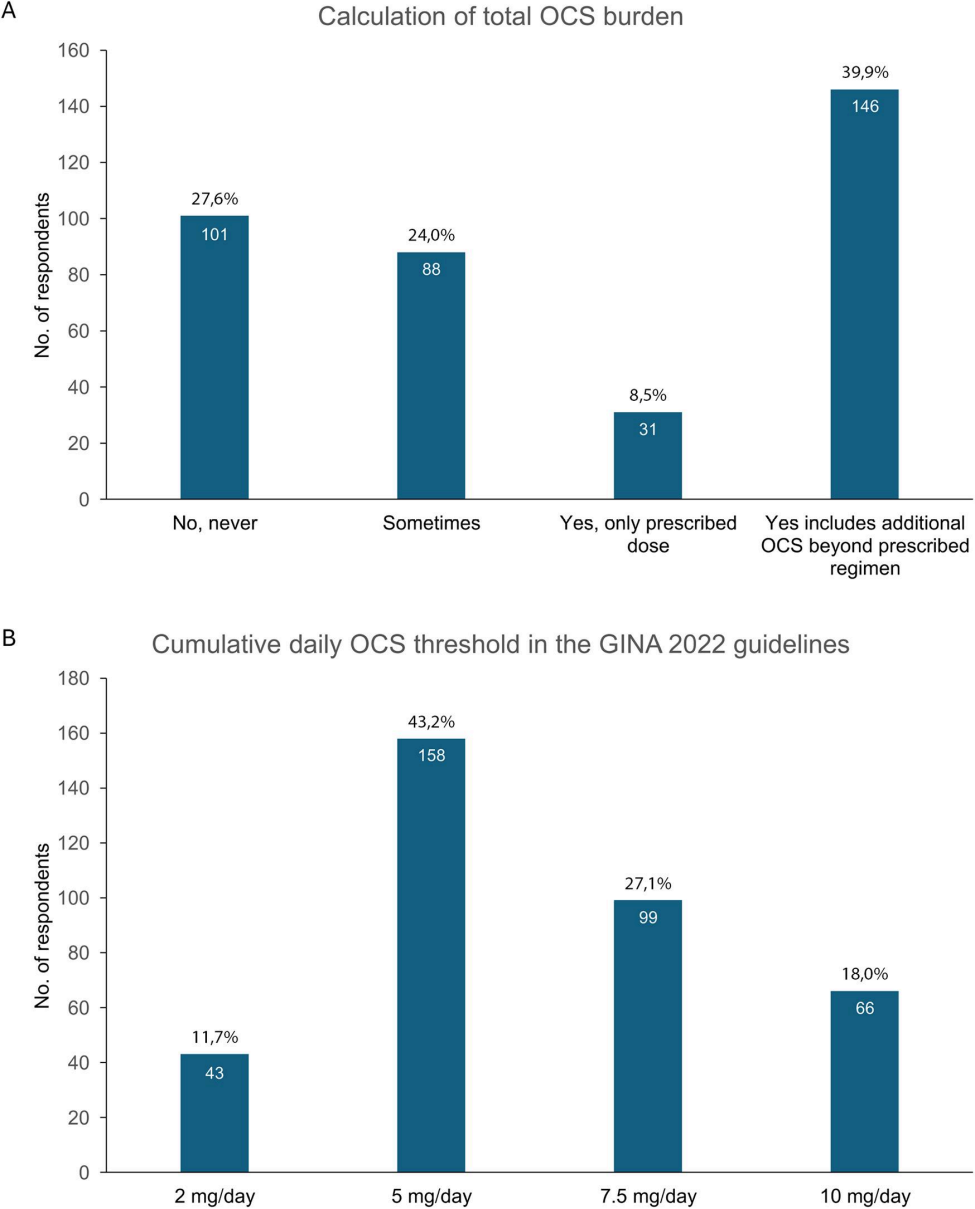
**(A)** Proportion of HCPs tracking the cumulative annual OCS dose, and **(B)** HCPs' perspectives on the maximum safe daily OCS dose. GINA, Global Initiative for Asthma; HCPs, healthcare professionals; OCS, oral corticosteroids.

Forty percent of HCPs reported keeping track of the total OCS dose for their patients. However, 28% reported never doing this, whereas 24% reported doing it only occasionally. Only 8% of HCPs based their calculation of the total OCS dose on the doses that had been prescribed (Figure 3A).

#### 3.1.4 S1.Q4: daily dose limitations for safety compliance

Question 4 asked HCPs to indicate the cumulative daily OCS dose that should not be exceeded according to the Global Initiative for Asthma (GINA) 2022 guidelines. The answers were quite heterogeneous. Forty-three percent of the respondents believed that the maximum safe daily dose was 5 mg/day, whereas 27% indicated 7.5 mg/day. The thresholds of 10 mg/day and 2 mg/day were reported by 18% and 12% of respondents, respectively (Figure 3B).

#### 3.1.5 S1.Q5: strategies to minimize oral corticosteroid use in severe asthma

**Figure 4:**
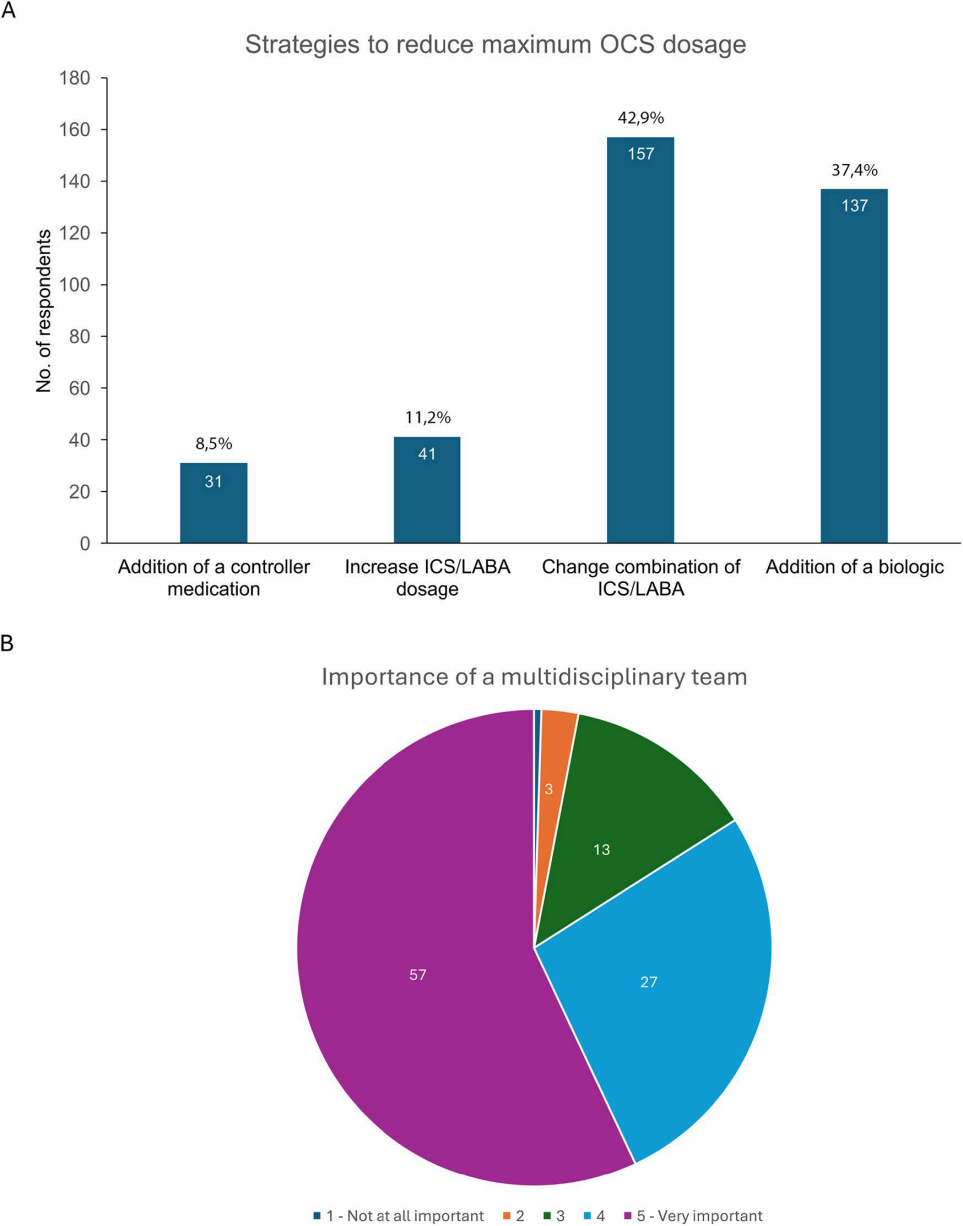
**(A)** Strategies preferred by HCPs to reduce OCS use in severe asthma management, and **(B)** perceptions of the importance of a multidisciplinary approach in preventing and managing the adverse effects of OCS. HCPs, healthcare professionals; ICS, inhaled corticosteroids; LABA, long-acting beta_2_-agonist; OCS, oral corticosteroids.

When asked to rank the strategies they use to minimize the OCS dose in managing SA, the most popular choice was to change the ICS/LABA combination (43%) followed by using biologic therapies (37%). Other answers were increasing the ICS/LABA dose (11%), or adding a controller drug (8%; Figure 4A).

#### 3.1.6 S1.Q6: importance of a multidisciplinary approach to prevent adverse effects

HCPs perceived that a multidisciplinary approach is very important (57%) or important (27%) in preventing and managing the negative effects of OCS, according to respondents. Less than 4% thought it was either not very important (3%) or not important at all (<1%), while 13% were neutral (Figure 4B).

#### 3.1.7 S1.Q7: frequency of monitoring oral corticosteroid-dependent patients

**Figure 5:**
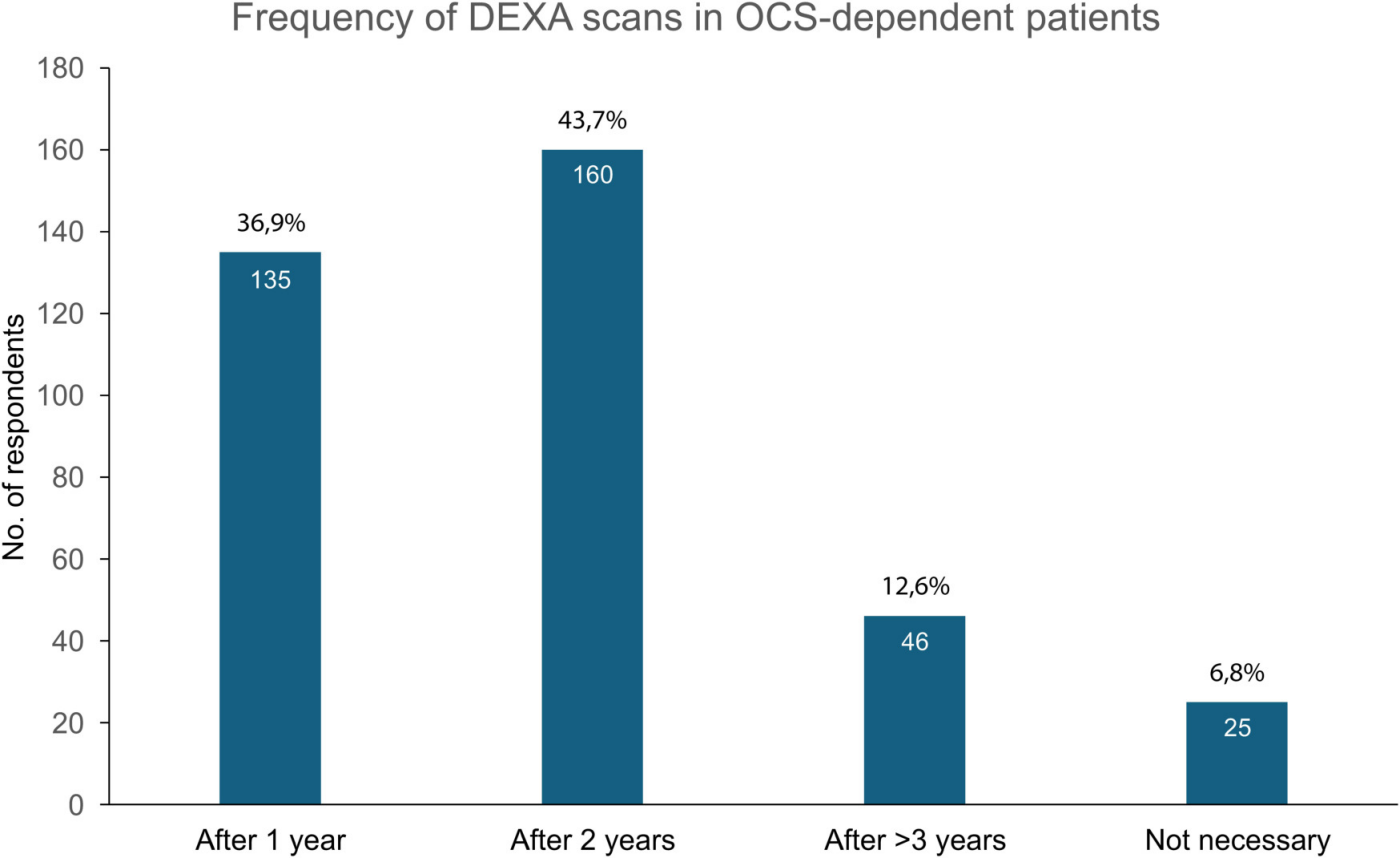
Frequency of monitoring visits for OCS-dependent patients. DEXA, dual-energy x-ray absorptiometry; OCS, oral corticosteroids.

Most HCPs recommend monitoring visits (MOC visits) for bone densitometry every two years (44%) or annually (37%). Smaller proportions of respondents suggested longer intervals (over 3 years, 12%) or indicated that MOC visits were unnecessary (Figure 5).

### 3.2 Survey 2—patients

Overall, 829 questionnaires were completed, 676 directly by patients with asthma and 153 by the family members or caregivers. Of the family members or caregivers who completed the survey, 75 were family members or guardians of adult patients and 78 cared for children with asthma.

#### 3.2.1 S2.Q1-Q3: respondent profile, severe asthma diagnosis and prescription history

The first three questions of Survey 2 were designed to establish the demographic characteristics of the respondents, to ensure that the respondents had a diagnosis of SA and to collect basic information regarding the respondents' history of OCS use for asthma. Of the total survey population, 361 patients had non-SA and 468 had SA (Table 1). The vast majority of patients with SA (95%) have been prescribed OCS, compared with approximately two-thirds of patients with non-SA (65%; Table 2).

**Table 1:** Respondent demographics and asthma condition breakdown for Survey 2.

Characteristics	Respondents *n* (%) (All patients *N* = 829)
Non-severe asthma	361 (44)
Severe asthma	468 (56)
Patients with asthma	676 (82)
Family members or caregivers of a patient with asthma	153 (18)
Family member of adult	61 (7)
Caregiver of adult	14 (2)
Family member of child	68 (8)
Caregiver of child	10 (1)

**Table 2:** Oral corticosteroid usage patterns and patient involvement in treatment decisions.

	Responses to questions, *n* (%)		
	Non-severe asthma (*n* = 361)	Severe asthma (*n* = 468)	All patients (*N* = 829)
Question 3: Have you ever been prescribed OCS for asthma			
Yes	234 (65)	445 (95)	679 (82)
No/I don't remember	127 (35)	23 (5)	150 (18)
Question 4: If yes, how long have you used them?			
I do not take/have not taken OCS	133 (37)	23 (5)	156 (19)
Every day of the year	48 (13)	131 (28)	179 (22)
For multiple consecutive days at least twice a year	99 (27)	220 (47)	319 (38)
I used to take them, but not anymore	51 (14)	60 (13)	111 (13)
Other	30 (8)	34 (7)	64 (8)
Question 5: Do you take OCS for asthma without a medical prescription?			
I do not take OCS	140 (39)	54 (12)	194 (23)
Once a year	22 (6)	56 (12)	78 (9)
Twice a year	16 (4)	55 (12)	71 (9)
More than three times a year	52 (14)	109 (23)	161 (19)
Never	131 (36)	194 (41)	325 (39)
Question 6: If you take OCS, do you taper the dose gradually when you stop?			
I do not take OCS	140 (39)	15 (3)	155 (19)
Yes, always	116 (32)	131 (28)	24 (30)
Yes, sometimes	1 (0)	110 (24)	111 (13)
No	112 (31)	185 (40)	297 (36)
Other	5 (1)	19 (4)	24 (3)
Question 7: Have you ever been involved by your doctor in the decision to take OCS?			
I do not take OCS	122 (34)	42 (9)	164 (20)
Yes	154 (43)	333 (71)	487 (59)
No	69 (19)	61 (13)	130 (16)
I don't remember	16 (4)	32 (7)	48 (6)

OCS, oral corticosteroids.

#### 3.2.2 S2.Q4: duration of oral corticosteroid use

About half of the patients with SA took OCS more than once for at least two cycles per year (47%), and 28% of patients use them daily (Table 2). In non-SA patients, these percentages are 27% and 13%, respectively. Overall, 14% of patients with non-SA and 13% with SA stated that they have ceased using OCS.

#### 3.2.3 S2.Q5: Non-prescribed use of oral corticosteroids

About one third of patients with non-SA (36%) and 41% of SA patients never used OCS without a prescription. On the other hand, 47% of patients with SA compared with 24% of patients with non-SA used OCS once a year or more without a prescription (Table 2).

#### 3.2.4 S2.Q6: dose tapering of oral corticosteroids

Approximately half of the patients with SA (52%) and one-third of those with non-SA (32%) always or sometimes taper the dose when stopping, whereas 40% of SA patients and 31% of the patients with non-SA do not (Table 2).

#### 3.2.5 S2.Q7: involvement in decision-making

Most patients reported that their physician involved them in the decision to take OCS, although this was more frequent among the SA than the non-SA patients (71% vs. 43%); approximately one-fifth of patients in both groups were not involved or could not recall being consulted in the decision (Table 2).

#### 3.2.6 S2.Q8: mention of alternatives

Among the non-SA patients, 45% were not informed about possible alternative therapies to OCS and 7% did not recall being given this information, while in the SA group, 49% were not offered alternatives; overall, 11% did not recall being offered alternatives (Table 3).

**Table 3:** Alternative treatments, specialist referrals and monitoring practices among asthma patients.

	Responses to questions, *n* (%)		
	Non-severe asthma (*n* = 361)	Severe asthma (*n* = 468)	All patients (*N* = 829)
Question 8: Has your doctor ever mentioned alternatives to OCS in your case			
I do not take OCS	126 (35)	33 (7)	159 (19)
Yes	47 (13)	141 (30)	188 (23)
No	164 (45)	227 (49)	391 (47)
I don't remember	24 (7)	67 (14)	91 (11)
Question 9: If used have used OCS for a long period, what strategy did your doctor adopt?			
I do not take OCS	155 943)	47 (10)	202 (24)
Added a new inhaler medication	74 (20)	105 (22)	179 (22)
Changed the inhaler medication	56 (16)	135 (29)	191 (23)
Added a biologic drug to the inhalation therapy	6 (2)	105 (22)	111 (13)
Changed the biologic drug to another one	2 (1)	8 (2)	10 (1)
Other	68 (19)	68 (15)	136 (16)
Question 10: Has your doctor referred you to other specialists to assess any potential side effects of prolonged OCS use?			
I do not take OCS	147 (41)	37 (8)	184 (22)
Yes	55 (15)	147 (31)	202 (24)
No	156 (43)	267 (57)	423 (51)
I don't remember	3 (1)	17 (4)	20 (2)
Question 11: Has the doctor treating your asthma ever explained the possible side effects of OCS?			
I do not take OCS	139 (39)	33 (7)	172 (21)
Yes	78 (22)	192 (41)	270 (33)
No	128 (35)	199 (43)	327 (39)
I don't remember	16 (4)	44 (9)	60 (7)
Question 12: If you are an asthma patient who has been taking oral corticosteroids for a long time, how often have you had an exam to assess the health of your bones? (DEXA = Bone Densitometry)			
I do not take OCS	163 (38)	61 (13)	224 (27)
Never	138 (38)	263 (56)	401 (48)
Once a year	51 (14)	133 (28)	184 (22)
Twice a year	9 (2)	8 (2)	17 (2)
More than twice a year	0	3 (1)	3 (0)
Question 13: When you buy corticosteroid tablets directly (to take orally) without a prescription, what is the main reason for doing so?			
I do not take OCS	9 (2)	7 (1)	16 (2)
To control asthma symptoms	42 (12)	104 (22)	146 (18)
Out of habit	0	0	0
Fear that my condition may worsen	36 (10)	104 (22)	140 (7)
I don't know	2 (1)	1 (0)	3 (0)
Other	4 (1)	4 (1)	8 (1)

OCS, oral corticosteroids.

#### 3.2.7 S2.Q9: long-term strategy

In the group without SA, changes (16%) or additions to inhaler medications (20%) were relatively common, while biologics were rarely added (2%) or changed (1%). In the SA group, more patients started on biologics (22%) or changed inhaler medications (29%; Table 3).

#### 3.2.8 S2.Q10-11: specialist referral and explanation of side effects

When asked about side effects of OCS, 22% of non-SA patients reported that they were informed about possible adverse reactions and 15% were referred to a specialist because of an OCS-related complication, whereas 35% reported that they were never informed, and 43% were not referred to another doctor to follow up on potential side effects. Among the SA patients, 43% reported that they were not informed while 57% reported that they were not referred to a specialist (Table 3). In this group, 41% were informed about potential side effects and 31% were referred to specialists for assessments of potential side effects.

#### 3.2.9 S2.Q12: bone health assessment

The majority of patients did not receive a bone health assessment (Table 3), but 16% of non-SA patients and 31% of SA patients reported undergoing bone densitometry at least once a year.

#### 3.2.10 S2.Q13: reason for purchasing oral corticosteroids without a prescription

A much higher proportion of SA than non-SA patients reported purchasing OCS without a prescription because they felt they needed it (Table 3). For instance, 22% of patients with SA turn to OCS to control their symptoms compared with 12% of patients with non-SA, and 22% of SA patients and 10% of non-SA patients purchase OCS because they are afraid their condition may worsen.

**Figure 6:**
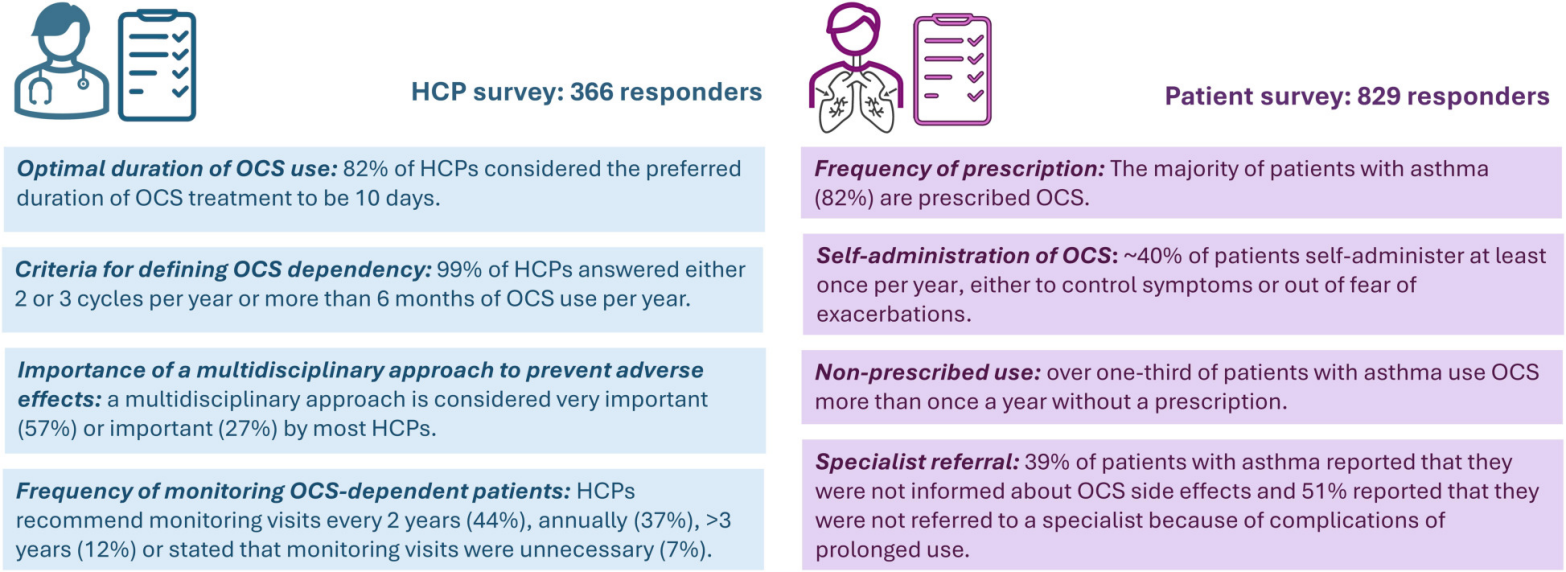
Key results of Survey 1 (HCPs) and Survey 2 (patients) regarding the prescription and monitoring of OCS for asthma. HCPs, healthcare professionals; OCS, oral corticosteroids.

The key results of Survey 1 and Survey 2 are summarized in Figure 6.

## 4 Discussion

In this study, we analyzed responses from two surveys, one aimed at HCPs and the other at patients, to understand their perceptions of the use of OCS in asthma treatment, especially in severe cases. These surveys were designed to gain insights into OCS use and clinical practices, which can then be used to improve the information available to both clinicians and patients.

These data show that prescription of OCS is common practice in Italy, and that general OCS use and frequent bursts of OCS are higher than recommended. As per the guidelines, OCS use should be minimized but more than 80% of all respondents, two-thirds of non-SA patients and almost all patients with SA (95%) received an OCS prescription. These data is consistent with other Italian studies which have shown that 64% of SA patients use OCS with doses exceeding 10 mg/day (18, 19).

First of all, our data suggest that there could be better communication between physicians and patients with regard to OCS use. Overall, 71% of SA patients reported being involved in decisions about OCS use, but only 43% of non-SA patients were involved in OCS decision-making. Patients also reported that their physicians did not explain the potential AEs of OCS (43% vs. 35% for SA and non-SA patients, respectively) nor did they suggest alternative treatments (49% vs. 45%, respectively).

HCPs tend to prescribe OCS bursts for approximately 10 days, which is considered to be clinically appropriate (20, 21), allowing discontinuation of the treatment without the need for tapering (8). Our findings are consistent with those of Busby and colleagues, who reported the median treatment duration to be 14 days with 40 mg of oral prednisolone based on analysis of clinical data from 61 studies and 1,608 patients (22). However, it is worth noting that, in our study, approximately one in five HCPs prescribed prolonged steroid courses (>21 days) to their patients. Understanding the rationale behind these clinical decisions is crucial to improving treatment practices. Defining a standardized treatment duration might be challenging (14), since there is no clear definition in the literature of what is a “short” course of treatment; thus, making comparisons between studies difficult. For example, some studies define courses shorter than 14 days (23) or less than 30 days (24) as short. From the clinician's perspective, the patient's history and response to treatment may play a key role in determining the optimal duration of treatment, but patients may view things differently. In the current survey, three out of four patients with SA take OCS at least twice per year, if not daily, but 40% of these patients do not or only occasionally taper the dose.

Guidelines support the use of OCS based on clinical need: the treatment of acute exacerbations requires the short-term use of OCS with a recommended duration of 3–5 days for children (6–11 years, 1–2 mg/kg/day up to 40 mg/day) and 5–7 days for adults, with doses between 40 and 50 mg/day (8). Long-term OCS should be used only in the management of SA as a last resort (GINA Step 5), with maintenance at low doses (≤7.5 mg/day). Dependence in phase 3 clinical trials is defined as OCS use for at least 6 months (25–28).

Frequent and prolonged use of OCS is a sign of uncontrolled asthma (29) and is associated with complications that may be related to either dependence or dosage (30). AEs include weight gain, diabetes, osteoporosis, glaucoma, anxiety, depression, cardiovascular disease, immunosuppression, and adrenal insufficiency (31–33). These AEs were identified in 93% of patients with SA (31). AEs can develop even during short-term use (<30 days) (34), and long-term use is associated with a higher risk of mortality compared to non-use (35). In Italy, the annual per-patient cost of OCS-related AEs is approximately €1960 in patients with asthma, almost double the cost associated with these events in patients without asthma (6). The authors suggested that approaches aimed at reducing OCS use in severe asthma, through the use of alternative treatments such as biologics, could minimize cost savings while improving patient outcomes. Guidelines also recommend asthma therapies such as biologics that improve symptom control while also reducing OCS dependence. The GINA guidelines recommend adding biologic drugs for SA patients at Step 5 before resorting to OCS and focus on the avoidance of maintenance OCS therapy (8).

The definition of OCS dependency and OCS burden depend on the criteria and clinical perspective used. Most major international organizations [GINA, Allergy Asthma Network [AAN], and European Respiratory Society/American Thoracic Society [ERS/ATS]] are consistent in defining asthma as uncontrolled if the patient has severe and frequent exacerbations which require at least two courses of OCS in a year (8, 36, 37). However, guidelines diverge when it comes to the definition of short- and long-term OCS use, contributing to considerable variation in how OCS burden is defined, depending on the frequency of OCS bursts (≥2 bursts annually) (37, 38), cumulative doses (0.5 to <1 g) (39), and duration of use (at least 6 months) (25–28). Although none of the guidelines specify temporal cut-offs of OCS use or for OCS-dependency, about two-thirds of HCPs in our study considered patients to be OCS-dependent if they needed two or three courses of OCS in a year. This suggests that, in real-world clinical settings, physicians might be aware of the risks associated with non-chronic steroid use.

Although the annual cumulative OCS dose is a marker of asthma control (20), the calculation of this parameter is not yet systematic or standardized. Approximately one-third of the HCPs in our survey never calculate the cumulative dose, and only 40% of HCPs consider all potential sources of corticosteroid exposure, including self-administered doses, which may result in underestimation of the overall risk of long-term OCS use. However, there is no clear and well-defined approach to the calculation of the total dose and there are no strict protocols to prevent steroid dependency. Calculating the cumulative OCS dose is complicated since patients do not always adhere to the prescribed dosage and regimen. Thus, there is a clear need for a standardized approach to the assessment of cumulative OCS exposure. There is also a need for a shared definition of “OCS dependency” that can be easily understood by both physicians and patients, and which would consistently identify the patients who would benefit from treatments other than OCS to control their symptoms. Greater clarity in the definition of OCS dependence could improve physician-patient communication and help reach a consensus on when to change the therapy, so that patients are neither over- nor under-treated.

A considerable number of respondents reported taking maximum daily cumulative OCS doses other than the 7.5 mg/day dose recommended by GINA guidelines, indicating variability in clinical practice and GINA guideline adherence. This may be due to a limited knowledge of the guidelines or the preference of the clinician based on their clinical experience. For instance, some literature suggests that the maximum OCS dose should not exceed 2.5 mg/day (40).

A daily dose lower than 7.5 mg/day offers some protection against potentially fatal adrenal suppression. However, doses equivalent to 2.5–7.5 mg/day of prednisone increase the risks of cardiovascular disease, severe infections, hypertension, diabetes, osteoporosis, fractures, and overall mortality, particularly in patients with type 2 diabetes (41). The use of ≥5 mg daily of corticosteroids for ≥3 months has been associated with a 50%–60% increase in the risk of osteoporotic fractures (42). In clinical practice, it is common to reduce the OCS dosage as much as possible, depending on the patient's risk profile, in order to minimize the risks associated with these medications. The lowest effective dose should be achieved and maintained, and whenever the guideline-recommended threshold is exceeded, measures should be taken to prevent complications such as bone fractures.

In cases of ongoing OCS therapy, the ERS/ATS guidelines recommend monitoring various parameters, including bone density, and using prophylactic measures to prevent bone density loss (37). The GINA guidelines suggest long-term risk-benefit assessment and patient monitoring for prevention of bone loss, encouraging referral to specialist care if ≥2 OCS courses are required in a year (8). Our data show a significant divergence of opinions among HCPs. While approximately four out of five HCPs are proactive in their approach and recommend a bone mineral density (BMD) test every 1–2 years, one in five believes the test is either not a priority or unnecessary (recommending it every 3 years or not at all). Nevertheless, the patient data show a different picture: 56% of the patients with SA (38% with non-SA) were never advised to undergo a BMD test, whereas 31% and 16% of the SA and non-SA patients, respectively, had BMD testing at least once per year.

Our results imply a need to improve awareness and the dissemination of GINA recommendations to promote consistent practices and improve the safety of OCS treatment. In Italy, the pharmacological management of asthma is suboptimal, with high use of OCS (19). In general the use of OCS has not declined (43), and patients are also being treated with increasingly higher OCS doses (14, 44, 45), up to approximately ten times those recommended by guidelines (8). To minimize the maximum OCS exposure, HCPs opt for switching ICS/LABA combinations or introducing biologics into the treatment regimen. In our survey, 11% of HCPs reported increasing the ICS/LABA dose, implying that some SA patients are receiving submaximal doses of ICS/LABAs. It is somewhat surprising that only a small proportion of HCPs prefers adding a controller to the prescription, but it could be justified if the patient with SA was already using an antimuscarinic agent. On a day-to-day basis, SA patients more frequently had their inhaler medication changed (29%) than had the addition of a new one or the prescription/change of a biologic (22% and 24%, respectively); therefore, the full treatment options recommended for SA patients in GINA (Step 5) are not being applied.

Similar results were reported in a study by Milger and colleagues (46), which focused on the use of OCS over biologics in Germany in 2019. These researchers also found that recommendations to prescribe biologics preferentially over OCS for SA were not followed as OCS were used more frequently (in 69% of cases in pulmonologists' practices) than biologics (37%) as add-on therapies in GINA 5 treatments. In the study by Milger and colleagues, 75% of patients with uncontrolled asthma remained at GINA 4 treatment level, often relying on OCS instead of biologics, even though the introduction of biologics was associated with a significant reduction in the use of short-acting β_2_-agonists (SABA) by 28%, high-dose OCS prescriptions by 55%, and overall OCS exposure by 40%. One-third of patients discontinued OCS entirely after starting biologics. On the contrary, patients treated in tertiary referral outpatient departments were more likely to receive biologics (66%) than those managed solely by a pulmonologist. Evidence-based guidelines significantly improve medical care and outcomes. However, their successful implementation is a complex process and depends on numerous factors, including the guidelines themselves, the broader social, cultural, and organizational contexts, and the characteristics of both physicians and patients, and must address barriers such as knowledge, attitudes, skills, experiences, beliefs and values of both physicians and patients (47).

Another key aspect in the management of SA is the identification and treatment of comorbidities and their impact on therapeutic choices. Most HCPs stated that multidisciplinary care plays a key role in preventing the side effects of OCS. To implement multidisciplinary care, the management of SA should involve several specialists, including otolaryngologists, endocrinologists, gastroenterologists, pulmonologists, allergists, and psychologists/psychiatrists. Nevertheless, 57% of patients with SA and 43% of those with non-SA are not referred to other specialists. A study of the emotional and psychological impact of OCS use in asthma patients found that the most common concerns were long-term side effects of OCS (91%) and weight gain (80%), but 67% of patients expressed concerns about becoming dependent on the medication and 73% expressed concerns about having to take OCS (48). Anxiety and depression are common comorbidities in patients with SA, contributing to poor quality of life and more frequent occurrences of dyspnea or disordered breathing (49). Psychological support may be particularly important for patients who take corticosteroids without a prescription due to fear of exacerbations, as this may be a sign of anxiety. If psychologists or psychiatrists are not available, trained HCPs could administer tests to identify patients in need of appropriate referrals. Lastly, alexithymia is a neglected but significant factor affecting between 9% and 63% of patients with asthma, potentially leading to a distorted perception of the disease and an underestimation of its severity (50). For example, an Italian study found that patients with higher levels of alexithymia had worse asthma control, as measured by the Asthma Control Test (ACT) (*r* = −0.31 *p* = 0.002), and health-related quality of life, and reported greater negative impacts of asthma and rhinitis on their daily lives (i.e., poorer management of asthma symptoms, including pain, nausea, fatigue, stiff joints, upset stomach and loss of strength) compared with patients who do not have alexithymia (50).

Of concern is that about 40% of patients self-administer OCS at least once per year, either to control symptoms or out of fear of exacerbations (51). In Italy, OCS are available as over-the-counter medicines that can be dispensed without a prescription. These data are consistent with research among Italian pharmacists which showed that 34.8% of clients asked for OCS without a prescription before the Coronavirus disease 2019 (COVID-19) pandemic and 43.9% have asked for OCS since the pandemic (51). In that survey, the conditions most frequently associated with requests for OCS without a prescription were upper airways (75.3%) or obstructive lung disease (68.1%). About two-thirds of individuals (63%–66%) who asked for OCS without a prescription were chronic users of OCS. The most common reasons for seeking OCS were for emergency use, because they had forgotten the prescription or had difficulty obtaining a prescription (51). The high rate of OCS use without a prescription (and the fact that some patients do so because they have difficulty obtaining a prescription) suggests that some patients take OCS without their physician's knowledge or against their physician's advice. The data highlight the need for targeted education for patients with asthma, particularly those with SA, focusing on the fact that exacerbations can be prevented by using inhaled therapy regularly, as well as the need for physicians to question patients regularly about their self-prescribed, over-the-counter OCS use.

### 4.1 Unmet needs

The data analysis allowed us to identify several unmet needs. The wide variability in clinical practice suggests that either knowledge of guidelines, or their application in clinical practice, remains inadequate. Better strategies are needed to ensure that HCPs not only know about these guidelines but are also equipped to follow them effectively. The lack of information about treatment pathways and the poor involvement of the patient in decision-making highlight the need for better patient education and empowerment. It is crucial to implement strategies that actively involve patients in shared decision-making to improve adherence and personalize care. A clearly defined and universally accepted definition of OCS dependency could help clinicians to diagnose dependency and prioritize alternative treatments. Inadequate access to multidisciplinary care hinders comprehensive patient management. Many patients are not referred to specialists for risk assessments regarding the long-term use of OCS. Overcoming barriers such as the limited availability of specialists or logistical challenges could improve patient outcomes, but this will require systemic changes at policy level. There is a clear unmet need for more systematic and frequent monitoring protocols, which could allow the identification of AEs at an early stage and reduce the risks associated with the prolonged use of OCS. Patient adherence and the actual steroid burden could be monitored using a clinical diary (tangible or digital) to track medication intake and daily symptom management. The diary could be complemented by the testing of clinical markers, including morning cortisol levels or bone density parameters, to obtain a more reliable estimate of patient adherence and treatment effects. Last but not least, there is the need for psychosocial support, which may entail involving mental health professionals in the asthma management team. This could address issues such as anxiety, treatment adherence, and quality of life for patients who self-medicate due to fear of exacerbations.

To answer some of these unmet needs, we propose the following targeted clinical and systemic intervention strategies: (1) Formulate a pragmatic definition of “OCS dependency” that is useful to both clinicians and patients, to avoid misunderstandings and inadequate practices; (2) develop reliable clinical markers and tools, such as morning cortisol levels and BMD assessment to monitor patients on OCS, identify adverse treatment effects and assess their compliance with the treatment regimen; (3) create a practical guide for long-term management of OCS-dependent patients so that HCPs have access to a practical handbook containing treatment strategies and practical indications based on the current best practices to ensure consistent and effective patient care; (4) develop patient monitoring apps tailored to patients' needs, to assist with adherence to treatment and help collect information on the patient's clinical status and treatment—these apps could include gamified elements to enhance engagement, as well as useful and practical reminders; and (5) invest in education programs for HCPs aimed at supporting clinical decision-making and standardizing clinical practices. This training should also include the use of new monitoring tools and guidelines on the optimal use of OCS.

### 4.2 Study limitations

The findings of our study must be considered in light of the following limitations. The lack of distinction between respondents from pediatric and adult care settings may lead to the misinterpretation of significant differences in OCS use between children and adults, and hinders our ability to make specific recommendations for patient groups with different requirements. The study was based on cross-sectional survey data, which may be subject to recall bias, response bias and inaccurate self-reporting, potentially impacting the reliability of the results, especially on the adherence to treatment and the frequency of OCS use. The survey was distributed to a specific set of Italian HCPs who were included in a proprietary database maintained by Sanofi Italia and patients, which introduces selection bias, and the study cohort may not represent the broader population of Italian asthma patients or practitioners, limiting the generalizability of the findings.

## 5 Conclusions

The results of our surveys show the diversity of approaches used to manage OCS use in asthma treatment in Italy. However, there is still a significant discrepancy between clinical practice and guideline recommendations, and the use of OCS remains very high. The differences in OCS prescribing patterns, treatment duration, and the recommended cumulative daily doses highlight the need for clearer guidelines and adherence to standardized practices. Patient participation in treatment decisions is still inadequate, emphasizing the need to improve communication between physicians and patients and enhance the shared decision-making process. These surveys have identified a range of unmet needs that can be addressed through initiatives to limit the use of OCS in patients with SA through better standardization of terminology and practices, improved access to multidisciplinary care, and ongoing education and support for HCPs and patients.

## Data Availability

The raw data supporting the conclusions of this article will be made available by the authors, without undue reservation.

## References

[1] GBD Chronic Respiratory Diseases Collaborators. Global burden of chronic respiratory diseases and risk factors, 1990–2019: an update from the global burden of disease study 2019. EClinicalMedicine. (2023) 59:101936. 10.1016/j.eclinm.2023.101936 37229504 PMC7614570

[2] Liu H Zhang J Liu L Lian G Shi R Xu M Global disease burden and attributable risk factor analysis of asthma in 204 countries and territories from 1990 to 2019. Allergy Asthma Immunol Res. (2023) 15:473–95. 10.4168/aair.2023.15.4.473 37153981 PMC10359648

[3] The global asthma report 2022. Int J Tuberc Lung Dis. (2022) 26:1–104. 10.5588/ijtld.22.1010 36303302

[4] Xing Y Jin Y Guo C Tam LS Wu D. Global, regional, and national trends in incidence of asthma: findings from the global burden of disease study 2019. Eur Respir J. (2023) 62:PA2074. 10.1183/13993003.congress-2023.pa2074

[5] Scortichini M Mennini FS Marcellusi A Paoletti M Tomino C Sciattella P. The economic burden of asthma in Italy: evaluating the potential impact of different treatments in adult patients with severe eosinophilic asthma. Eur J Health Econ. (2024) 26:869–876. 10.1007/s10198-024-01736-5 39690320 PMC12204866

[6] Canonica GW Colombo GL Bruno GM Di Matteo S Martinotti C Blasi F Shadow cost of oral corticosteroids-related adverse events: a pharmacoeconomic evaluation applied to real-life data from the Severe Asthma Network in Italy (SANI) registry. World Allergy Organ J. (2019) 12:100007. 10.1016/j.waojou.2018.12.001 30937132 PMC6439414

[7] Menzies-Gow A Hoyte FL Price DB Cohen D Barker P Kreindler J Clinical remission in severe asthma: a pooled *post hoc* analysis of the patient journey with benralizumab. Adv Ther. (2022) 39:2065–84. 10.1007/s12325-022-02098-1 35287231 PMC9056458

[8] Global Initiative for Asthma. Global Strategy for Asthma Management and Prevention, 2024. Accessed May 2024, (2024).

[9] Campbell RG. Risks and management of long-term corticosteroid use in chronic rhinosinusitis. Curr Opin Otolaryngol Head Neck Surg. (2018) 26:1–7. 10.1097/MOO.0000000000000421 29059082

[10] Brennan V Martin-Grace J Greene G Heverin K Mulvey C McCartan T The contribution of oral and inhaled glucocorticoids to adrenal insufficiency in asthma. J Allergy Clin Immunol Pract. (2022) 10:2614–23. 10.1016/j.jaip.2022.05.031 35697207

[11] Cutrera R Baraldi E Indinnimeo L Miraglia Del Giudice M Piacentini G Scaglione F Management of acute respiratory diseases in the pediatric population: the role of oral corticosteroids. Ital J Pediatr. (2017) 43:31. 10.1186/s13052-017-0348-x 28335827 PMC5364577

[12] Ekstrom M Nwaru BI Hasvold P Wiklund F Telg G Janson C. Oral corticosteroid use, morbidity and mortality in asthma: a nationwide prospective cohort study in Sweden. Allergy. (2019) 74:2181–90. 10.1111/all.13874 31095758 PMC6899917

[13] Lee H Ryu J Nam E Chung SJ Yeo Y Park DW Increased mortality in patients with corticosteroid-dependent asthma: a nationwide population-based study. Eur Respir J. (2019) 54:1900804. 10.1183/13993003.00804-2019 31515404

[14] Bleecker ER Menzies-Gow AN Price DB Bourdin A Sweet S Martin AL Systematic literature review of systemic corticosteroid use for asthma management. Am J Respir Crit Care Med. (2020) 201:276–93. 10.1164/rccm.201904-0903SO 31525297 PMC6999108

[15] Hew M McDonald VM Bardin PG Chung LP Farah CS Barnard A Cumulative dispensing of high oral corticosteroid doses for treating asthma in Australia. Med J Aust. (2020) 213:316–20. 10.5694/mja2.50758 32906192 PMC7589219

[16] Eger K Amelink M Hashimoto S Hekking PP Longo C Bel EH. Overuse of oral corticosteroids, underuse of inhaled corticosteroids, and implications for biologic therapy in asthma. Respiration. (2022) 101:116–21. 10.1159/000518514 34535586

[17] Jaffuel D Fabry-Vendrand C Darnal E Wilczynski O Pain E Bourdin A. Perception of oral corticosteroids in adult patients with asthma in France. J Asthma. (2021) 58:946–57. 10.1080/02770903.2020.1748048 32285714

[18] Heffler E Blasi F Latorre M Menzella F Paggiaro P Pelaia G The severe asthma network in Italy: findings and perspectives. J Allergy Clin Immunol Pract. (2019) 7:1462–68. 10.1016/j.jaip.2018.10.016 30368004

[19] Latorre M Rizzi A Paggiaro P Baiardini I Bagnasco D Del Giacco S Asthma management, focused on the use of oral corticosteroids: the opinions of Italian asthmatic patients. J Asthma. (2024) 61:1294–305. 10.1080/02770903.2024.2338863 38578082

[20] Suehs CM Menzies-Gow A Price D Bleecker ER Canonica GW Gurnell M Expert consensus on the tapering of oral corticosteroids for the treatment of asthma. A delphi study. Am J Respir Crit Care Med. (2021) 203:871–81. 10.1164/rccm.202007-2721OC 33112646

[21] Fuhlbrigge AL Lemanske RF Jr Rasouliyan L Sorkness CA Fish JE. Practice patterns for oral corticosteroid burst therapy in the outpatient management of acute asthma exacerbations. Allergy Asthma Proc. (2012) 33:82–9. 10.2500/aap.2012.33.3499 22183118

[22] Busby J Khoo E Pfeffer PE Mansur AH Heaney LG. The effects of oral corticosteroids on lung function, type-2 biomarkers and patient-reported outcomes in stable asthma: a systematic review and meta-analysis. Respir Med. (2020) 173:106156. 10.1016/j.rmed.2020.106156 32979621

[23] Aljebab F Choonara I Conroy S. Systematic review of the toxicity of short-course oral corticosteroids in children. Arch Dis Child. (2016) 101:365–70. 10.1136/archdischild-2015-309522 26768830 PMC4819633

[24] Price D Castro M Bourdin A Fucile S Altman P. Short-course systemic corticosteroids in asthma: striking the balance between efficacy and safety. Eur Respir Rev. (2020) 29:190151. 10.1183/16000617.0151-2019 32245768 PMC9488828

[25] Rabe KF Nair P Brusselle G Maspero JF Castro M Sher L Efficacy and safety of dupilumab in glucocorticoid-dependent severe asthma. N Engl J Med. (2018) 378:2475–85. 10.1056/NEJMoa1804093 29782224

[26] Bel EH Wenzel SE Thompson PJ Prazma CM Keene ON Yancey SW Oral glucocorticoid-sparing effect of mepolizumab in eosinophilic asthma. N Engl J Med. (2014) 371:1189–97. 10.1056/NEJMoa1403291 25199060

[27] Nair P Wenzel S Rabe KF Bourdin A Lugogo NL Kuna P Oral glucocorticoid-sparing effect of benralizumab in severe asthma. N Engl J Med. (2017) 376:2448–58. 10.1056/NEJMoa1703501 28530840

[28] Wechsler ME Colice G Griffiths JM Almqvist G Skarby T Piechowiak T SOURCE: a phase 3, multicentre, randomized, double-blind, placebo-controlled, parallel group trial to evaluate the efficacy and safety of tezepelumab in reducing oral corticosteroid use in adults with oral corticosteroid dependent asthma. Respir Res. (2020) 21:264. 10.1186/s12931-020-01503-z 33050928 PMC7550846

[29] Haughney J Winders T Holmes S Chanez P Menzies-Gow A Kocks J A charter to fundamentally change the role of oral corticosteroids in the management of asthma. Adv Ther. (2023) 40:2577–94. 10.1007/s12325-023-02479-0 37027115 PMC10080509

[30] Henderson I Caiazzo E McSharry C Guzik TJ Maffia P. Why do some asthma patients respond poorly to glucocorticoid therapy? Pharmacol Res. (2020) 160:105189. 10.1016/j.phrs.2020.105189 32911071 PMC7672256

[31] Sweeney J Patterson CC Menzies-Gow A Niven RM Mansur AH Bucknall C Comorbidity in severe asthma requiring systemic corticosteroid therapy: cross-sectional data from the optimum patient care research database and the British thoracic difficult asthma registry. Thorax. (2016) 71:339–46. 10.1136/thoraxjnl-2015-207630 26819354

[32] Sullivan PW Ghushchyan VH Globe G Schatz M. Oral corticosteroid exposure and adverse effects in asthmatic patients. J Allergy Clin Immunol. (2018) 141:110–16 e7. 10.1016/j.jaci.2017.04.009 28456623

[33] Hyland ME Whalley B Jones RC Masoli M. A qualitative study of the impact of severe asthma and its treatment showing that treatment burden is neglected in existing asthma assessment scales. Qual Life Res. (2015) 24:631–9. 10.1007/s11136-014-0801-x 25201169

[34] Waljee AK Rogers MA Lin P Singal AG Stein JD Marks RM Short term use of oral corticosteroids and related harms among adults in the United States: population based cohort study. Br Med J. (2017) 357:j1415. 10.1136/bmj.j1415 28404617 PMC6284230

[35] Bleecker ER Al-Ahmad M Bjermer L Caminati M Canonica GW Kaplan A Systemic corticosteroids in asthma: a call to action from world allergy organization and respiratory effectiveness group. World Allergy Organ J. (2022) 15:100726. 10.1016/j.waojou.2022.100726 36582404 PMC9761384

[36] Allergy & Asthma Network. What is severe asthma?, (2025).

[37] Chung KF Wenzel SE Brozek JL Bush A Castro M Sterk PJ International ERS/ATS guidelines on definition, evaluation and treatment of severe asthma. Eur Respir J. (2014) 43:343–73. 10.1183/09031936.00202013 24337046

[38] Asthma and Allergy Foundation of America. Oral Corticosteroid Stewardship Statement, (2018).

[39] Price DB Trudo F Voorham J Xu X Kerkhof M Ling Zhi Jie J Adverse outcomes from initiation of systemic corticosteroids for asthma: long-term observational study. J Asthma Allergy. (2018) 11:193–204. 10.2147/JAA.S176026 30214247 PMC6121746

[40] Canonica GW Blasi F Paggiaro P Senna G Passalacqua G Spanevello A Oral corticosteroid sparing with biologics in severe asthma: a remark of the Severe Asthma Network in Italy (SANI). World Allergy Organ J. (2020) 13:100464. 10.1016/j.waojou.2020.100464 32999699 PMC7509464

[41] Beuschlein F Else T Bancos I Hahner S Hamidi O van Hulsteijn L European society of endocrinology and endocrine society joint clinical guideline: diagnosis and therapy of glucocorticoid-induced adrenal insufficiency. J Clin Endocrinol Metab. (2024) 109:1657–83. 10.1210/clinem/dgae250 38724043 PMC11180513

[42] Takhar JS Pawar VK Marneris AG Doan TA Acharya NR Gonzales JA. When to consider bisphosphonates in patients on steroids for chronic ocular inflammatory conditions. J Ocul Pharmacol Ther. (2019) 35:379–80. 10.1089/jop.2019.0058 31241403 PMC7141554

[43] Tran TN King E Sarkar R Nan C Rubino A O'Leary C Oral corticosteroid prescription patterns for asthma in France, Germany, Italy and the UK. Eur Respir J. (2020) 55:1902363. 10.1183/13993003.02363-2019 32165402 PMC7270349

[44] Volmer T Effenberger T Trautner C Buhl R. Consequences of long-term oral corticosteroid therapy and its side-effects in severe asthma in adults: a focused review of the impact data in the literature. Eur Respir J. (2018) 52:1800703. 10.1183/13993003.00703-2018 30190274

[45] Vetrano DL Zucchelli A Bianchini E Marconi E Lombardo FP Cricelli C Patterns of oral corticosteroids use in primary care patients with severe asthma. Respir Med. (2020) 166:105946. 10.1016/j.rmed.2020.105946 32250874

[46] Milger K Koschel D Skowasch D Timmermann H Schmidt O Bergmann KC Maintenance OCS were used more frequently than biologics in patients with uncontrolled GINA 4/5 asthma in Germany in 2019. J Asthma Allergy. (2024) 17:1093–101. 10.2147/JAA.S480380 39502931 PMC11536981

[47] Baiardini I Braido F Bonini M Compalati E Canonica GW. Why do doctors and patients not follow guidelines? Curr Opin Allergy Clin Immunol. (2009) 9:228–33. 10.1097/ACI.0b013e32832b4651 19390434

[48] Cooper V Metcalf L Versnel J Upton J Walker S Horne R. Patient-reported side effects, concerns and adherence to corticosteroid treatment for asthma, and comparison with physician estimates of side-effect prevalence: a UK-wide, cross-sectional study. NPJ Prim Care Respir Med. (2015) 25:15026. 10.1038/npjpcrm.2015.26 26158805 PMC4497315

[49] Stubbs MA Clark VL Gibson PG Yorke J McDonald VM. Associations of symptoms of anxiety and depression with health-status, asthma control, dyspnoea, dysfunction breathing and obesity in people with severe asthma. Respir Res. (2022) 23:341. 10.1186/s12931-022-02266-5 36510255 PMC9743554

[50] Silvestro O Ricciardi L Catalano A Vicario CM Tomaiuolo F Pioggia G Alexithymia and asthma: a systematic review. Front Psychol. (2023) 14:1221648. 10.3389/fpsyg.2023.1221648 37609491 PMC10441120

[51] Nappi E Keber E Paoletti G Casini M Group S Carosio C Oral corticosteroid abuse and self-prescription in Italy: a perspective from community pharmacists and sales reports before and during the COVID-19 era. J Pers Med. (2023) 13:833. 10.3390/jpm13050833 37241004 PMC10220562

